# Analysis of factors influencing the network teaching effect of college students in a medical school during the COVID-19 epidemic

**DOI:** 10.1186/s12909-021-02825-2

**Published:** 2021-07-23

**Authors:** Liang Yu, Long Huang, Hao-ru Tang, Na Li, Ting-ting Rao, Die Hu, Yu-feng Wen, Liu-xia Shi

**Affiliations:** 1 School of Humanities and Management, Wannan Medical College, Wuhu, 241002 Anhui China; 2 Teaching Quality Assessment Centre, Wannan Medical College, Wuhu, 241002 Anhui China; 3 School of Anesthesiology, Wannan Medical College, Wuhu, 241002 Anhui China; 4 Youth League Committee, Wannan Medical College, Wuhu, 241002 Anhui China; 5 Department of Nutrition and Food Hygiene, School of Public Health, Wannan Medical College, Wuhu, 241002 Anhui China; 6 Department of Oral Medicine, School of Stomatology, Wannan Medical College, Wuhu, 241002 Anhui China

**Keywords:** Network teaching, Effect, College students, COVID-19

## Abstract

**Background:**

The purpose of this study is to understand the influencing factors of Chinese college students’ satisfaction with online teaching and psychological pressure on learning during the novel coronavirus epidemic.

**Methods:**

We assessed the effect of online teaching of 7084 medical students from wannan medical college in March 5 to April 2, 2020 using cluster sampling. The respondents were asked to complete a 7-item self-compiled online teaching satisfaction questionnaire. Chi-square test and multivariate logistic regression analysis are used.

**Results:**

Sex is female (*OR* = 1.257, 95%*CI*: 1.132 ~ 1.396), grades are second and third grades (second grades: *OR* = 1.228, 95%*CI*: 1.080 ~ 1.397; third grades: *OR* = 1.197, 95%*CI*: 1.048 ~ 1.367), normal/unfamiliar learning platform operation (*OR* = 3.692, 95%*CI*: 3.321 ~ 4.103) were risk factors for satisfactory teaching effect. In addition, students whose school year system is four-year (*OR* = 0.870, 95%*CI*: 0.781 ~ 0.969) and grade 4 and above (*OR* = 0.594, 95%*CI*: 0.485 ~ 0.727) were more satisfied with the teaching effect of teachers. And, during the period of the COVID-19 epidemic, the risk factors for college students to have psychological stress were: female (*OR* = 1.258, 95%*CI*: 1.096 ~ 1.442), from rural areas (*OR* = 1.511, 95%*CI*: 1.312 ~ 1.740), and the academic year system is four-year system (*OR* = 1.191, 95%*CI*: 1.028 ~ 1.380), using mobile phones and other learning tools (*OR* = 1.388, 95%CI: 1.205 ~ 1.600), general/unfamiliar with learning platform operations (*OR* = 2.273), 95%*CI*: 1.888 ~ 2.735). While the protective factors for college students’ psychological stress included: grade three and four and above (*OR* = 0.463, 95%*CI*: 0.387 ~ 0.554; *OR* = 0.232, 95%*CI*: 0.187 ~ 0.286), and they think that the teaching effect is satisfactory (*OR* = 0.314, 95%*CI*: 0.261 ~ 0.379).

**Conclusion:**

This survey shows that compared with male college students, female college students were more dissatisfied with the teaching effect of teachers and havd greater psychological pressure on learning. Psychological counseling should be strengthened for students in rural areas and those who were not familiar with the operating platform to relieve their psychological pressure on learning.

**Supplementary Information:**

The online version contains supplementary material available at 10.1186/s12909-021-02825-2.

## Background

Coronavirus disease 2019 (COVID-19) is a disease caused by SARS-CoV-2 (severe acute respiratory syndrome coronavirus 2) that causes respiratory infections [1]. Since December 2019, the COVID-19 epidemic has spread rapidly in China and around the world, arousing worldwide attention [2]. As of April 19, 2021, about 14 million people were affected by COVID-19, and 3,020,688 people died as a result [3]. In addition to the main transmission routes such as droplet transmission and close contact transmission, COVID-19 is also transmitted by the virus in the aerosol when the patient is in the same closed space, and the virus is also present in the stool and urine of the patient [4]. Because the transmission route of COVID-19 is respiratory tract and strong transmission intensity, the measures of “resident isolation at home” were used by China to control the spread of the epidemic. In order to ensure the safety of students during the epidemic and the continued study of students. China, Anhui Provincial Department of Education issued a notice on February 24, 2020: Various schools in Anhui Province (high schools, vocational schools, primary and secondary schools, kindergartens, etc.) postponed the start of school, and implemented online education and teaching notice on March 2, 2020 [5].

In the era of Internet development, online teaching has long been proposed. In 2015, in China, the Ministry of Education put forward the “Opinions on Strengthening the Construction and Application of Online Open Courses in Colleges and Universities” to further promote the development of online teaching [6]. Therefore, online teaching methods had been skillfully used during the epidemic. However, due to the particularity of class time, the effect of teachers’ online teaching and the factors that affect students’ online learning efficiency were still uncertain.

Teachers teaching effectiveness and student satisfaction with teaching is a two-way process of influence. In the teaching process, whether there is communication between teachers and students has a great impact on the teaching effect. A meta-analysis by Kyaw et al. [7] found that the communication between medical students and teachers had an impact on online teaching effectiveness and student satisfaction. Thirty-nine schools and 20 students of otolaryngology were surveyed by Offergeld [8] and others. It was found that the network teaching equipment was not perfect and the communication between medical teachers and students had a great influence on the teaching effect and the students’ satisfaction with the teacher’s teaching. In a meta-analysis, it was discovered that factors such as students’ self-learning effectiveness and electronic devices used in learning have an impact on students’ learning satisfaction [9]. College students are more likely to have psychological problems such as stress and anxiety when faced with the dual pressure of the epidemic and online learning [10].

However, so far, there are few studies on the effect of online teaching and psychological stress during the COVID-19 epidemic period. In order to understand the current situation of online teaching in colleges and universities, students’ satisfaction with online teaching and their psychological pressure during the COVID-19 epidemic period. From the perspective of students, we investigated the students’ satisfaction with the way of online learning and the teaching effect of teachers, as well as the obstacles and psychological pressure in online learning in a medical college during the epidemic period. The current research will help to find and solve the problems of online teaching in time, and truly implement effective learning education for students, which is of great significance to alleviate students’ psychological pressure, improve teaching level and improve the quality of applied talents training.

## Methods

### Study population and sample

The target population comprised undergraduates of wannan medical college. The respondents in the target population were sampled by cluster sampling. We assessed the effect of online teaching of these students during the COVID-19 outbreak by using structured questionnaires. The questionnaires were anonymous to ensure the confientiality and reliability of data. Finally, 7084 respondents that completed the questionnaires were included in the final analysis (100% response rate), and, 2990 were males, accounting for 42.21%; 4094 were females, accounting for 57.79%. They volunteered to participate in this study and signed an online informed consent form before collecting data. This study was approved by the Academic Ethics Committee of Wannan Medical College.

### Instruments

The study instrument comprised a structured questionnaire packet that inquired demographic information, including sex, grade, major, online teaching platform and proficiency, among others. They were also asked to complete a 7-item online teaching satisfaction questionnaire adapted from the questionnaire used in Qin et al. (2020)‘s study [11] (see S1 File 1), which mainly measures college students’ satisfaction with teaching methods, arrangements, effect, psychological pressure, access to teaching schedule information, and teacher’s preparation. Satisfaction with the teaching effect is divided into 5 levels: 1 = very satisfied, 2 = satisfied, 3 = relatively satisfied, 4 = fair, 5 = dissatisfied. There are 5 levels of whether there is psychological pressure: 1 = no pressure at all, 2 = a little pressure, 3 = a certain amount of pressure, 4 = a lot of pressure. In this survey, the number of people who was satisfied with the teaching effect was the sum of the people who was very satisfied, relatively satisfied, and satisfied. The number of people with psychological pressure was the sum of the number of people with pressure, a certain amount of pressure, and a lot of pressure. The results of reliability analysis showed that the Cronbach’s Alpha of the scale was 0.873, and the test-retest reliability coefficient was 0.757. The results of exploratory factor analysis showed that KMO = 0.849, the cumulative variance explained 64.724% of the total variance, and the content validity index was 0.830, indicating that the scale had good reliability and validity.

### Data analysis

Data were analyzed with SPSS Version 26.0. We used the chi-square test to conduct a general descriptive analysis of the influencing factors of college students’ online teaching satisfaction. Statistically significant variables were screened and included in multivariate logistic regression analyses. The estimates of the strengths of associations were demonstrated by the odds ratio (OR) with a 95% confidence interval (CI). The collinearity test showed that the Tolerance was far greater than 0.1, and the variance inflation factor (VIF) was less than 10, suggesting that there was no collinearity among the variables. Multivariate Logistic regression analysis method was used for multivariate analysis. In univariate analysis, the method of *P* < 0.1 variables into the multivariate Logistic regression and the method of variable selection was Stepwise. A two-tailed *P* < 0.05 was considered statistically significant. The assignment of variables is shown in Table 1.

**Table 1:** Assignment of research variables

Variable	Assignment
Sex	1 = Male; 2 = Female
Area	1 = Town; 2 = Rural area
Length of schooling	1 = Five-year; 2 = Four-year
Grade	1 = First grade; 2 = Second grade; 3 = Third grade; 4 = Grade 4 and above
Network tools used	1 = Computer/tablet; 2 = Mobile phone and others
Proficiency	1 = Proficiency; 2 = General / unskilled

## Results

### The effect of online teaching and psychological pressure in different sexes, regions, length of schooling and grades

During the COVID-19 epidemic, the satisfaction of female college students with teachers’ preparatory preparation were higher than that of male college students (*P* < 0.05), and the satisfaction of male college students with their pre-class preparation and teaching effects were higher than that of female college students (*P* < 0.05). The psychological pressure of male college students in learning were lower than that of female college students (*P*<0.05). The university students from urban areas were higher than those from rural students (*P*<0.05) in preparation for their own classes, timely access to information on teaching arrangements, teaching methods and arrangements, answers to questions and answers, and satisfaction with teaching results. The psychological pressure on learning from urban college students were lower than that from rural college students (*P*<0.05). Undergraduates with a five-year academic system had lower psychological stress in learning than undergraduates with a four-year academic system (*P*<0.05), while non-student students have to prepare for teachers, prepare for themselves, and obtain teaching in time. The satisfaction of students of different grades with the teacher’s pre-class preparation, the satisfaction with the student’s pre-class preparation, the satisfaction of the students with timely information about the teaching arrangement, the satisfaction with the teacher’s teaching methods and arrangements, the satisfaction with the doubts being resolved, and the teachers’ satisfaction with teaching effect is different(*P*<0.05). There were differences in the learning psychological pressure of students in different grades(*P*<0.05). See Table 2.

**Table 2:** During the epidemic period, college students’ online teaching effectiveness and psychological pressure were compared among the basic characteristics of students (n/%)

Item	Satisfaction with teacher’s preparation	Satisfaction with students’ preparation	Timely access to teaching schedule information satisfaction	Satisfaction with teaching methods and arrangements	Satisfaction with getting answers	Satisfaction with teaching effect	Psychological pressure
Sex							
Female (*n* = 2990)	2793 (93.4)	2473 (82.7)	2525 (84.4)	2152 (72.0)	2586 (86.5)	2007 (67.1)	2451 (82.0)
Male (*n* = 4094)	3895 (95.1)	3150 (76.9)	3466 (84.7)	2903 (70.9)	3476 (84.9)	2569 (62.8)	3526 (86.1)
χ^2^	9.775	35.108	0.060	0.958	3.510	14.452	22.602
*P*	0.002	0.000	0.807	0.328	0.061	0.000	0.000
Area							
Town (*n* = 3533)	3345 (94.7)	2892 (81.9)	3038 (86.0)	2584 (73.1)	3092 (87.5)	2354 (66.6)	2863 (81.0)
Rural area (*n* = 3551)	3343 (94.1)	2731 (76.9)	2953 (83.2)	2471 (69.6)	2970 (83.6)	2222 (62.6)	3114 (87.7)
χ^2^	0.965	26.495	10.867	10.938	21.588	12.733	59.537
*P*	0.326	0.000	0.001	0.000	0.000	0.000	0.000
Length of schooling							
Five-year (*n* = 4262)	4014 (94.2)	3391 (79.6)	3628 (85.1)	3036 (71.2)	3657 (85.8)	2715 (63.7)	3517 (82.5)
Four-year (*n* = 2822)	2674 (94.8)	2232 (79.1)	2363 (83.7)	2019 (71.5)	2405 (85.2)	1861 (65.9)	2460 (87.2)
χ^2^	1.061	0.230	2.512	0.080	0.465	3.737	27.871
*P*	0.303	0.632	0.113	0.777	0.495	0.053	0.000
Grade							
First grade (*n* = 2508)	2388 (95.2)	1975 (78.7)	2093 (83.5)	1811 (72.2)	2139 (85.3)	1630 (65.0)	2252 (89.8)
Second grade (*n* = 1967)	1840 (93.5)	1534 (78.0)	1616 (82.2)	1329 (67.6)	1656 (84.2)	1201 (61.1)	1756 (89.3)
Third grade (*n* = 1902)	1783 (93.7)	1492 (78.4)	1633 (85.9)	1355 (71.2)	1644 (86.4)	1200 (63.1)	1504 (79.1)
Grade 4 and above (*n* = 707)	677 (95.8)	622 (88.0)	649 (91.8)	560 (79.2)	623 (88.1)	545 (77.1)	465 (65.8)
χ^2^	9.911	35.885	41.894	36.065	8.075	61.053	317.743
*P*	0.019	0.000	0.000	0.000	0.044	0.000	0.000

### The effect and psychological pressure of online teaching using different online teaching tools and proficiency

During the COVID-19 epidemic, college students who used computers/tablets as learning tools had higher satisfaction than those who used mobile phones as learning tools, in terms of preparation before class, timely access to teaching information arrangements, teaching methods of teachers, problems that can be solved, and teaching effects of teachers (all *P* < 0.05). College students who use computers/tablets as e-learning tools have lower psychological stress in learning than those who use mobile phones as e-learning tools (*P*<0.05). The satisfaction of college students with skilled operation of the learning platform were higher than that of college students with general/unfamiliar operation of the learning platform, in terms of pre-class preparation for teachers, their own pre-class preparation, timely access to teaching arrangement information, teachers’ teaching methods and arrangements, questions being answered, teaching effect, etc. (all *P* < 0.05). However, the students who were proficient in the operation of the learning platform in terms of psychological pressure on learning were lower than the college students who were generally/unfamiliar with the operation of the learning platform (*P* < 0.05). (See Table 3.)

**Table 3:** Comparison of online teaching effects and psychological pressure among college students using different online teaching tools and proficiency during the epidemic(n/%)

Item	Satisfaction with teacher’s preparation	Satisfaction with students’ preparation	Timely access to teaching schedule information satisfaction	Satisfaction with teaching methods and arrangements	Satisfaction with getting answers	Satisfaction with teaching effect	Psychological pressure
Network tools used							
Computer/tablet (*n* = 2474)	2334 (94.3)	2042 (82.5)	2150 (86.9)	1834 (74.1)	2150 (86.9)	1654 (66.9)	1980 (80.0)
Mobile phone and others (*n* = 4610)	4354 (94.4)	3581 (77.7)	3841 (83.3)	3221 (69.9)	3912 (84.9)	2922 (63.4)	3997 (86.7)
χ^2^	0.034	23.224	15.857	14.303	5.453	8.483	54.333
*P*	0.854	0.000	0.000	0.000	0.020	0.004	0.000
Proficiency							
Proficiency (*n* = 4751)	4612 (97.1)	4099 (86.3)	4389 (92.4)	3873 (81.5)	4322 (91.0)	3537 (74.4)	3805 (80.1)
General / unskilled (*n* = 2333)	2076 (89.0)	1524 (65.3)	1602 (68.7)	1182 (50.7)	1740 (74.6)	1039 (44.5)	2172 (93.1)
χ^2^	194.044	419.615	674.301	728.844	340.389	612.169	200.883
*P*	0.000	0.000	0.000	0.000	0.000	0.000	0.000

### Influential factors of teaching effect and harvest satisfaction

During the COVID-19 epidemic, the sex was female (*OR* = 1.257, 95%*CI*: 1.132 ~ 1.396), the grades were second and third grade (*OR* = 1.228, 95%*CI*: 1.080 ~ 1.397; *OR* = 1.197, 95%*CI*: 1.048 ~ 1.367), general/unfamiliar with the operation of the learning platform (*OR* = 3.692, 95%*CI*: 3.321 ~ 4.103) were the risk factors for college students to think that the teaching effect was satisfactory, the school year was a four-year system (*OR* = 0.870, 95%*CI*: 0.781 ~ 0.969), grade 4 and above (*OR* = 0.594, 95%*CI*: 0.485 ~ 0.727) were the protective factors for college students to think that the teaching effect was satisfactory. (See Table 4.)

**Table 4:** Analysis of influencing factors of college students’ teaching effect and harvest satisfaction during the epidemic

Item	*B*	*S.E.*	*Wald*	*P*	*OR*	*95%CI*	
Sex							
Male					1.000		
Female	0.229	0.054	18.269	0.000	1.257	1.132	1.396
Length of schooling							
Five-year					1.000		
Four-year	−0.139	0.055	6.466	0.011	0.870	0.781	0.969
Grade							
First grade					1.000		
Second grade	0.206	0.066	9.817	0.002	1.228	1.080	1.397
Third grade	0.179	0.068	7.008	0.008	1.197	1.048	1.367
Grade 4 and above	−0.521	0.103	25.579	0.000	0.594	0.485	0.727
Proficiency							
Proficiency					1.000		
General/unskilled	1.306	0.054	586.001	0.000	3.692	3.321	4.103
Constant	−2.611	0.145	324.567	0.000			

### Factors influencing psychological stress

During the New Coronary Pneumonia epidemic, the risk factors for undergraduates’ psychological stress were: sex is female (*OR* = 1.258, 95%*CI*: 1.096 ~ 1.442), from rural areas (*OR* = 1.511, 95%*CI*:1.312 ~ 1.740), school year four-year system (*OR* = 1.191, 95%*CI*: 1.028 ~ 1.380), use of mobile phones and others as learning tools (*OR* = 1.388, 95%*CI*: 1.205 ~ 1.600), general/unfamiliar operation of the learning platform (*OR* = 2.273, 95%*CI*: 1.888 ~ 2.735). (See Table 5.)

**Table 5:** Influencing factors of college students’ learning psychological pressure during the epidemic

Item	*B*	*S.E.*	*Wald*	*P*	*OR*	*95%CI*	
Sex							
Male					1.000		
Female	0.229	0.070	10.722	0.001	1.258	1.096	1.442
Area							
Town					1.000		
Rural area	0.413	0.072	32.738	0.000	1.511	1.312	1.740
Length of schooling							
Five-year					1.000		
Four-year	0.175	0.075	5.401	0.020	1.191	1.028	1.380
Grade							
First grade					1.000		
Second grade	−0.037	0.101	0.131	0.717	0.964	0.791	1.175
Third grade	−0.770	0.091	70.944	0.000	0.463	0.387	0.554
Grade 4 and above	−1.463	0.108	182.999	0.000	0.232	0.187	0.286
Network tools used							
Computer/tablet					1.000		
Mobile phone and others	0.328	0.072	20.571	0.000	1.388	1.205	1.600
Proficiency							
Proficiency					1.000		
General/unskilled	0.821	0.095	75.443	0.000	2.273	1.888	2.735
Satisfactory teaching effect							
No					1.000		
Yes	−1.158	0.095	148.004	0.000	0.314	0.261	0.379
Constant	0.265	0.250	1.120	0.290			

## Discussion

Since COVID-19 outbreak, postpone the opening was selected to control the development of the epidemic of school. During the suspension period, colleges and universities had chosen online teaching. In order to understand the effect of online teaching during the epidemic, an online questionnaire survey was conducted on students in a medical school.

The result of this survey found that female college students were the risk factors that college students think they were satisfied with teaching. One thousand two hundrend students from some universities in Jiangxi, China were surveyed by Wang Xinxin [12], the results of the survey showed that the satisfaction of female students with the teaching effect of teachers were lower than that of male students, which was consistent with the results of this study. However, some studies had found that female students were more satisfied with the overall grade and teaching satisfaction than male students [13]. Female college students’ dissatisfaction with the teacher’s teaching effect during the epidemic may be related to the fact that female were more concerned about the teacher’s various behaviors, and more critical to the teacher. And the lower satisfaction of self-learning gains in female students may be related to female’s lower self-study efficacy and lower trust in their learning ability [14]. It may also be that females were more susceptible to environmental stressors, and males were more confident in overcoming the epidemic than girls. In addition, males were relatively rational and thick-lined, and havd a relatively positive attitude, they havd greater confidence and hope for the future. During the epidemic, females were more likely to be affected, which affects their online learning effects and reduces their satisfaction with teaching. The results of this survey showed that, compared with first-year students, second-year students and third-year students were risk factors for satisfaction with teachers’ teaching effectiveness and their own gains, however the students in the fourth grade and above were the protective factors compared with the first grade. Compared with first-year students, sophomores and juniors were more familiar with the learning styles during college. Moreover, freshman students have only one semester of study time in the school due to the epidemic situation. Many freshmen follow the study habits of high school. They take classes more seriously and have a more respectful attitude towards teachers. Therefore, the freshman students were more satisfied with the teacher’s teaching effect and their own gains. Compared with first-year students, seniors and above students had greater self-control, and their sense of learning efficiency were higher, they realized that their future work and life are closely related to their current learning, and believe that online teaching can meet relevant needs, so students’ sense of self-learning efficiency will increase, so their satisfaction were higher than that of first-year students [15]. And this study found that general/unfamiliar learning platform operation is a risk factor for college students to think that teachers’ teaching effects are satisfactory. Students’ general/unfamiliar learning platform operation causes students’ nervousness and anxiety during their studies, as well as computer anxiety. As a result, the learning ability will be lowered, and the satisfaction with teaching will be reduced [16]. The study also found that the four-year school year was a protective factor for college students to think they were satisfied with the teaching effect of teachers. Compared with five-year students, four-year students have lower learning pressure, were more comfortable with online courses, have good learning effects, so they had high satisfaction with teaching effects.

This study not only investigated the satisfaction of students with online teaching during the epidemic, but also investigated the influencing factors of psychological stress in college students’ learning. The result showed that female college students were more likely to have psychological pressure on learning than male college students, the possible reason was that they have higher requirements for themselves and higher expectations for academic performance [17]. Therefore, education departments and colleges and universities should focus on caring for females and reducing stress. Teachers should guide females to use positive thinking to suppress stress generation and look at online learning with a positive perspective. College students from rural areas were more prone to psychological stress in learning. In rural areas, the network speed is slow, the video playback is stuck, and the data upload is slow. All these will affect the students’ perception of class and the students’ mood of learning, making students feel frustrated. Because of the network, students in rural areas were worried about the efficiency of online learning, and parents of rural students have high expectations for their learning, so their greater psychological stress [18, 19]. Students who used mobile phones as learning tools were more likely to have psychological pressure in learning. It may be because students using mobile phones gave lectures online when the teacher switches the mobile phone to other software, which missed some learning points, after the class, they felt anxious about not learning the content, resulting in psychological pressure. Undergraduates with general/unfamiliar learning platform operations were prone to psychological stress when learning. The possible reason was that they were unfamiliar with the operation of the learning platform, when they were learning online, they were anxious and worried due to fear of operation errors. Therefore, before the start of teaching, training students on the learning platform can further help students quickly master the learning software, adapt to the online learning method, and then be more satisfied with the online teaching effect.

Moreover, the study found that senior college students and self-satisfied teaching results were the protective factors for college students’ psychological stress in learning. With the increase of grades, the risk of undergraduates having psychological stress in learning was smaller, the possible reason was that the adaptability and mental endurance of senior college students had improved. And senior students have a higher level of understanding of epidemic prevention and control, which may be due to the fact that the older they are, the more experienced they are, the more peaceful the mentality, the relatively high environmental adaptability and psychological resistance to frustration, and the relatively objective and rational view of things. However, the results of this study are inconsistent with those of Aktekin et al. [20]. University students who were satisfied with the teaching effect were less likely to have psychological pressure on learning, indicating that satisfaction with the teaching effect will cause people to have positive emotions and be full of motivation for learning. Therefore, they had less psychological pressure.

Like other studies, there are some deficiencies in this survey. First of all, this research is a cross-sectional survey, which cannot verify the relationship between cause and effect. Secondly, this survey is an online survey, and there may be some deficiencies in the quality control of the questionnaire, which will affect the research results.

## Conclusion

In general, during the epidemic period, female students, students of grade two and grade three, students who were not familiar with the operation of learning platform were more dissatisfied with the teaching effect, while students with four-year academic year system and grade four or above were more satisfied with the teaching effect of teachers. The risk factors for college students’ psychological stress included: female, from rural areas, four-year academic year, using mobile phones and other learning tools, general/unfamiliar learning platforms. And the protective factors for college students’ psychological stress included: grades were third and fourth grades and above, think that the teaching effect was satisfactory.

## Supplementary Information

**Additional file 1.**

## Data Availability

The datasets generated and/or analysed during the current study are available from the corresponding author on reasonable request.

## Abbreviations

COVID-19: Coronavirus disease 2019
SARS-CoV-2: Severe acute respiratory syndrome coronavirus 2
OR: Odds ratio
CI: Confidence interval
VIF: Variance inflation factor
