# Supplementary material for: Analysis of factors influencing the network teaching effect of college students in a medical school during the COVID-19 epidemic
Source: BMC Med Educ. 2021 Jul 23;21:397. doi: 10.1186/s12909-021-02825-2 (PMC8300986; doi:10.1186/s12909-021-02825-2)
Supplement: Supplementary file 1 — Additional file 1. [file 12909_2021_2825_MOESM1_ESM.docx]

**Supporting information**

**S1 File 1.**

Guidance: Please answer according to your own true feelings during the COVID-19. 1= "Yes", 2= "Not".

I am satisfied with teacher's preparation

I am satisfied with my own study preparation

I am satisfied with timely access to teaching schedule information

I am satisfied with teaching methods and arrangements

I am satisfied with getting answers

I am satisfied with the teaching effect

I have psychological pressure on online teaching
